# The quantitative surface analysis of an antioxidant additive in a lubricant oil matrix by desorption electrospray ionization mass spectrometry

**DOI:** 10.1002/rcm.6690

**Published:** 2013-10-01

**Authors:** Caitlyn Da Costa, James C Reynolds, Samuel Whitmarsh, Tom Lynch, Colin S Creaser

**Affiliations:** 1 Centre for Analytical Science, Department of Chemistry, Loughborough UniversityLoughborough, LE11 3TU, UK; 2 Castrol, Technology CentreWhitchurch Hill, Pangbourne, Reading, RG8 7QR, UK

## Abstract

**RATIONALE:**

Chemical additives are incorporated into commercial lubricant oils to modify the physical and chemical properties of the lubricant. The quantitative analysis of additives in oil-based lubricants deposited on a surface without extraction of the sample from the surface presents a challenge. The potential of desorption electrospray ionization mass spectrometry (DESI-MS) for the quantitative surface analysis of an oil additive in a complex oil lubricant matrix without sample extraction has been evaluated.

**METHODS:**

The quantitative surface analysis of the antioxidant additive octyl (4-hydroxy-3,5-di-*tert*-butylphenyl)propionate in an oil lubricant matrix was carried out by DESI-MS in the presence of 2-(pentyloxy)ethyl 3-(3,5-di-*tert*-butyl-4-hydroxyphenyl)propionate as an internal standard. A quadrupole/time-of-flight mass spectrometer fitted with an in-house modified ion source enabling non-proximal DESI-MS was used for the analyses.

**RESULTS:**

An eight-point calibration curve ranging from 1 to 80 µg/spot of octyl (4-hydroxy-3,5-di-*tert*-butylphenyl)propionate in an oil lubricant matrix and in the presence of the internal standard was used to determine the quantitative response of the DESI-MS method. The sensitivity and repeatability of the technique were assessed by conducting replicate analyses at each concentration. The limit of detection was determined to be 11 ng/mm^2^ additive on spot with relative standard deviations in the range 3–14%.

**CONCLUSIONS:**

The application of DESI-MS to the direct, quantitative surface analysis of a commercial lubricant additive in a native oil lubricant matrix is demonstrated. © 2013 The Authors. *Rapid Communications in Mass Spectrometry* published by John Wiley & Sons, Ltd.

Desorption electrospray ionization (DESI) is an ambient ionization technique that enables the direct analysis of molecular analytes from surfaces with minimal sample preparation. DESI utilizes an electrospray directed at the surface to desorb and ionize analytes at atmospheric pressure resulting in electrospray ionization (ESI)-like mass spectra. Since the development of DESI in 2004,^1^ it has become one of the most commonly used techniques for the qualitative surface analysis of target analytes, and has been applied to the detection of a wide range of compounds desorbed from a variety of surface materials.^1^–^7^ The use of DESI mass spectrometry (DESI-MS) in quantitative analyses remains relatively unexplored, but has been reported for the analysis of complex matrices such as biological fluids,^8^,^9^ foodstuffs,^10^,^11^ polymers,^12^ and cosmetic formulations,^13^ showing the potential of the technique.

Lubricants form an essential component within tribological systems, i.e. systems where interacting surfaces move in relative motion, such as engines. Common commercial lubricants are formulated from a range of base fluids, either mineral or synthetic oils, in which chemical additives are dissolved. The base oil formulation and nature of the chemical additives will influence the physical and chemical properties of the lubricant and alter its potential applications.^14^ The use of atmospheric pressure ionization techniques for the qualitative mass spectrometric analysis of base oils and oil distillates has been demonstrated.^15^ Such techniques include atmospheric pressure photoionization,^16^ matrix-assisted laser desorption/ionization (MALDI),^17^ direct analysis in real time,^18^ ESI^19^ and DESI.^20^,^21^

Chemical additives are used in the development of lubricants for specific functions and can increase the life span of the product. The analysis of additives provides information regarding additive age, composition and degradation of the lubricant and many commercial lubricant additives have been studied by gas chromatography (GC) and ESI.^22^–^25^ One group of additives is antioxidants, which are commonly sterically hindered phenols or aromatic amines.^14^ The presence of oxygen and elevated temperatures within a tribological environment can cause rapid oxidation of lubricants, resulting in variation of the viscosity and acidity, beyond acceptable ranges, and in the generation of undesired breakdown products such as sludge. The analysis of lubricant antioxidant additives has been demonstrated using ESI-MS and MALDI-MS,^26^–^28^ but there have been no reports of the application of DESI-MS to the quantitative analysis of additives present in a native oil lubricant matrix. In this paper, we report the application of DESI-MS for the direct, quantitative analysis of a commercial lubricant antioxidant additive in a complex and native base oil matrix using a custom-built DESI source designed to enable non-proximal DESI-MS surface analysis.

## EXPERIMENTAL

### Reagents and chemicals

Methanol and water were purchased from Fisher Scientific (Loughborough, UK) and hexane was purchased from Sigma Aldrich (Gillingham, UK). All solvents were HPLC grade. The base oil matrix (group one treated base oil) and an antioxidant additive octyl (4-hydroxy-3,5-di-*tert*-butylphenyl)propionate (**I**) were supplied by Castrol (Pangbourne, UK) for the analysis. Ethylene glycol monopentyl ether and concentrated sulphuric acid were purchased from Sigma Aldrich and 3,5-di-*tert*-butyl-4-hydroxyphenylpropionic acid was purchased from Alfa Aesar (Heysham, UK) for the in-house synthesis of the internal standard.

### DESI-MS equipment and experimental conditions

**Figure 1:**
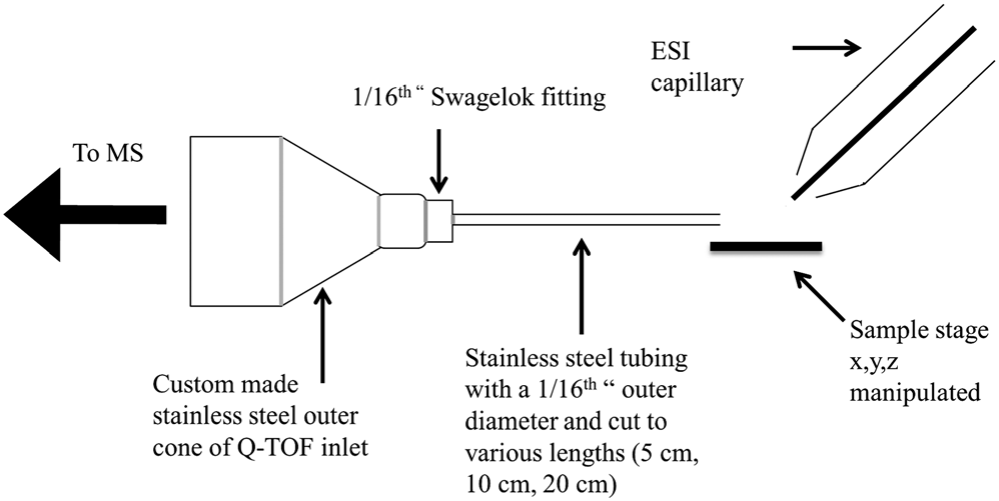
Schematic diagram of the modified inlet of the quadrupole/time-of-flight mass spectrometer for non-proximal DESI-MS analysis.

The DESI-MS analysis was conducted on a Waters Synapt HDMS quadrupole-time-of-flight (Q-TOF) mass spectrometer (Waters, Manchester, UK) which consists of a quadrupole, trap, T-wave ion mobility and transfer stacked-ring ion guide regions and a time-of-flight mass analyser. The instrument was equipped with an in-house modified DESI source equipped with a custom-built outer cone and x,y,z-manipulated sample stage. A schematic diagram of the DESI-MS source is shown in Fig. 1. The custom-built outer stainless steel cone fits over the standard inner cone of the inlet to the z-spray interface of the spectrometer, replacing the standard outer cone. A stainless steel Swagelok tube fitting (part number: SS-100-R-2, Swagelok, Manchester, UK) was welded to the cone enabling attachment of stainless steel extender tubing (1/16" (0.16 cm) o.d., 0.05 cm i.d., length 5–20 cm), which acts as an ion transfer tube for non-proximal DESI analyses.

The electrospray capillary was positioned at an angle of approximately 45° relative to the sample surface with an ESI tip to sample distance of ∼3 mm. The sample was positioned horizontally to the mass spectrometer inlet with an inlet to sample distance of ∼1 mm. Each sample was analysed in negative ion mode using an electrospray solvent of 95:5 MeOH/H_2_O at a flow rate of 20 μL/min. The instrumental parameters were: capillary voltage, –3 kV; sampling cone voltage, 20 V; source temperature, 120°C; desolvation gas (N_2_) flow rate, 100 L/h; and trap collision energy, 6 eV.

Structural confirmation of **I** in the oil matrix was conducted using DESI-MS/MS. Isolation of the precursor ion was achieved in the quadrupole and fragmentation was induced in the trap region using a trap collision energy of 36 eV. The observed product ion spectrum was compared with one obtained with a standard sample of **I** spotted in methanol onto a filter paper surface.

### Synthesis of internal standard (II)

The internal standard 2-(pentyloxy)ethyl 3-(3,5-di-*tert*-butyl-4-hydroxyphenyl)propionate (**II**) was synthesised via a Fischer esterification reaction. Ethylene glycol monopentyl ether (100 μL) and 3,5-di-*tert*-butyl-4-hydroxyphenylpropionic acid (47 mg) were mixed in a HPLC vial and concentrated H_2_SO_4_ (1 μL) was added as a catalyst. A pierced lid was fixed onto the vial to enable water to escape from the reaction mixture as steam, and the sample vortexed. The reaction vial was then heated to 105°C for 16 h.

### Sample preparation

Stock solutions were prepared by dissolving known weights of **I** (0.5–40 mg) in 1 mL hexane and spiking in 10 μL of **II** to give a nominal concentration of 13 mg/mL **II**. An aliquot of each standard solution (100 μL) was mixed with the base oil (400 μL). The resulting oil (10 μL) was spotted onto a filter paper surface to give deposited amounts of additive in the range of 1–80 µg of **I** per spot. The filter paper was secured onto a glass slide for support and positioned under the electrospray capillary on the sample stage. The sample was traversed under the electrospray in a horizontal motion perpendicular to the mass spectrometer inlet using the x,y,z-manipulator and data was acquired for a total of 1.5 min. A blank analysis was conducted before and after each sample to check for carryover. Six replicate analyses were conducted at each concentration of **I**. The sensitivity of the method was determined by calculating the limit of detection (LOD) from the absolute response of **I**.

## RESULTS AND DISCUSSION

**Figure 2:**
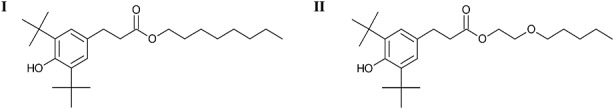
Structures of octyl (4-hydroxy-3,5-di-*tert*-butylphenyl)propionate (**I**), a commercial antioxidant additive, and 2-(pentyloxy)ethyl 3-(3,5-di-*tert*-butyl-4-hydroxyphenyl)propionate (**II**) internal standard.

The quantitative surface analysis of a commercial lubricant antioxidant additive, octyl (4-hydroxy-3,5-di-*tert*-butylphenyl)-propionate (**I**), in a complex base oil matrix by DESI-MS was carried out in negative ion mode using 2-(pentyloxy)ethyl 3-(3,5-di-*tert*-butyl-4-hydroxyphenyl)propionate (**II**) as an internal standard, to generate the deprotonated molecules ([M–H]^–^) of the target analytes. The structures of **I** and **II** are shown in Fig. 2. The modified DESI ion source (Fig. 1) was found to have improved sensitivity compared with the standard spectrometer configuration, because the custom-built outer cone enables a closer proximity of the mass spectrometer inlet to the sample surface. The cone assembly also allows mass spectrometer sampling to be carried out away from the front of the mass spectrometer, through the use of stainless steel ion transfer tubes, which can be used for the non-proximal DESI-MS analysis of larger objects.

**Figure 3:**
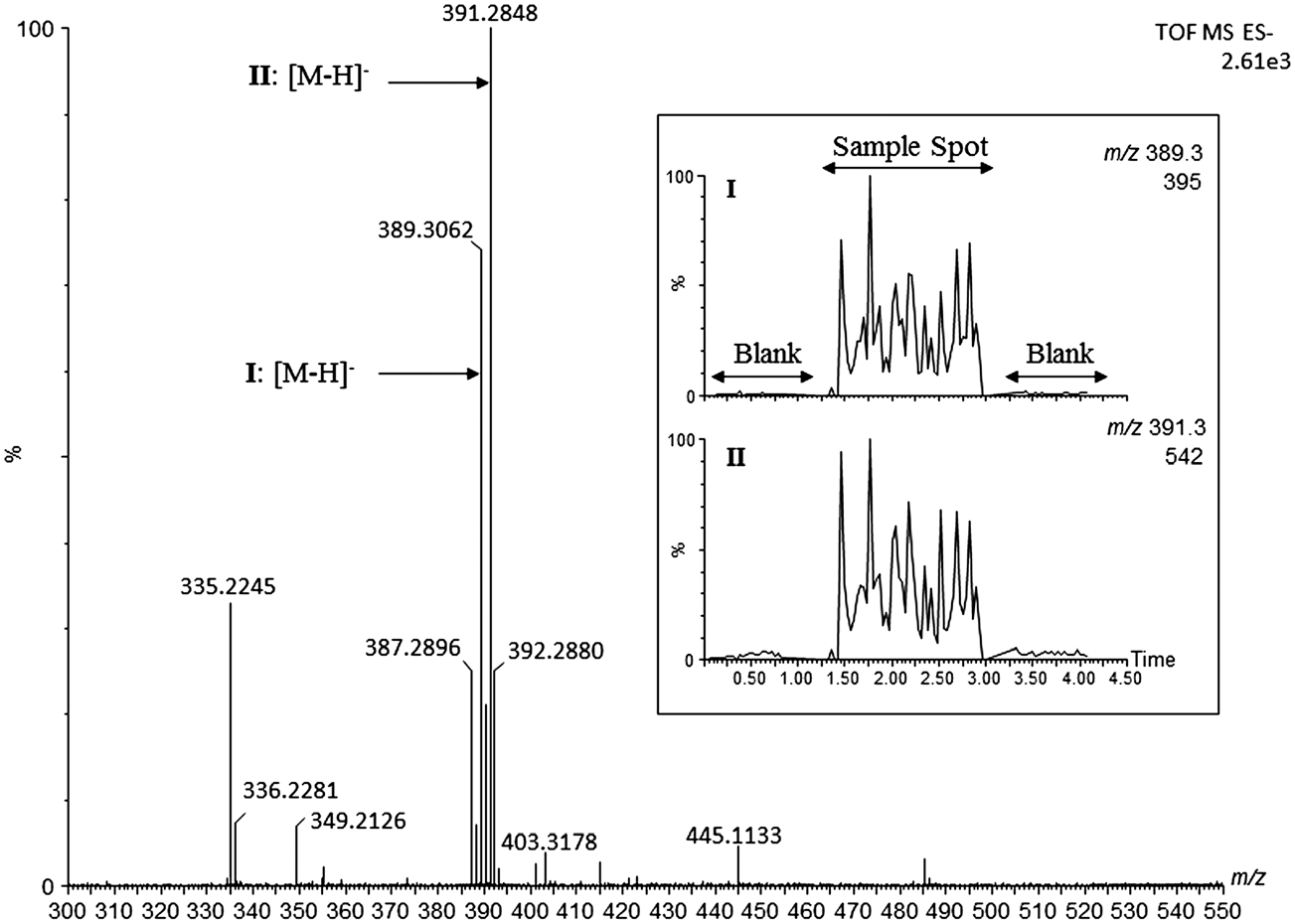
Mass spectrum showing the deprotonated molecules of **I** (10 µg) and **II** spiked into an oil matrix, spotted onto filter paper and analyzed by DESI-MS in negative ion mode. Insert: selected ion responses of the [M–H]^–^ ions of **I** (*m*/*z* 389.3) and **II** (*m*/*z* 391.3) for the DESI-MS analysis of a single spot.

The characterization of base oils and the detection of additives have been reported using both polar and non-polar electrospray phases with differing detection capabilities.^21^ The use of a polar electrospray phase, 95:5 MeOH/H_2_O, and negative mode analysis for the detection of the additive **I** in a base oil matrix using DESI-MS was found to generate a mass spectrum that had little chemical noise resulting from the base oil matrix. Figure 3 shows the DESI-MS spectrum obtained from the desorption of a spot containing **I** (10 µg) and the internal standard **II** (27 µg) in a base oil matrix deposited on a filter paper surface. Desorption from glass, PTFE and metal surfaces is also possible (data not shown). The deprotonated molecule of the additive at *m*/*z* 389 (observed 389.3062, calculated 389.3056, 1.5 ppm error) and the internal standard (*m*/*z* 391) can be clearly distinguished from the chemical noise resulting from the oil matrix. The insert in Fig. 3 shows the selected ion responses for the deprotonated molecules of **I**, *m*/*z* 389.3, and **II**, *m*/*z* 391.3, with the lubricant spot positioned under the electrospray to generate a DESI response for the sample. Blank areas of the surface were measured before and after the sample analysis, demonstrating the absence of background interference and sample-to-sample carry over. The analyte and internal standard responses began to decrease as a result of depletion of the sample from the surface and the sample spot was moved under the electrospray so that a new area of the spot could be analyzed. This process was repeated for the 1.5-min acquisition time, covering a cross-section of the spot, which was a representation of the whole sample deposited.

**Figure 4:**
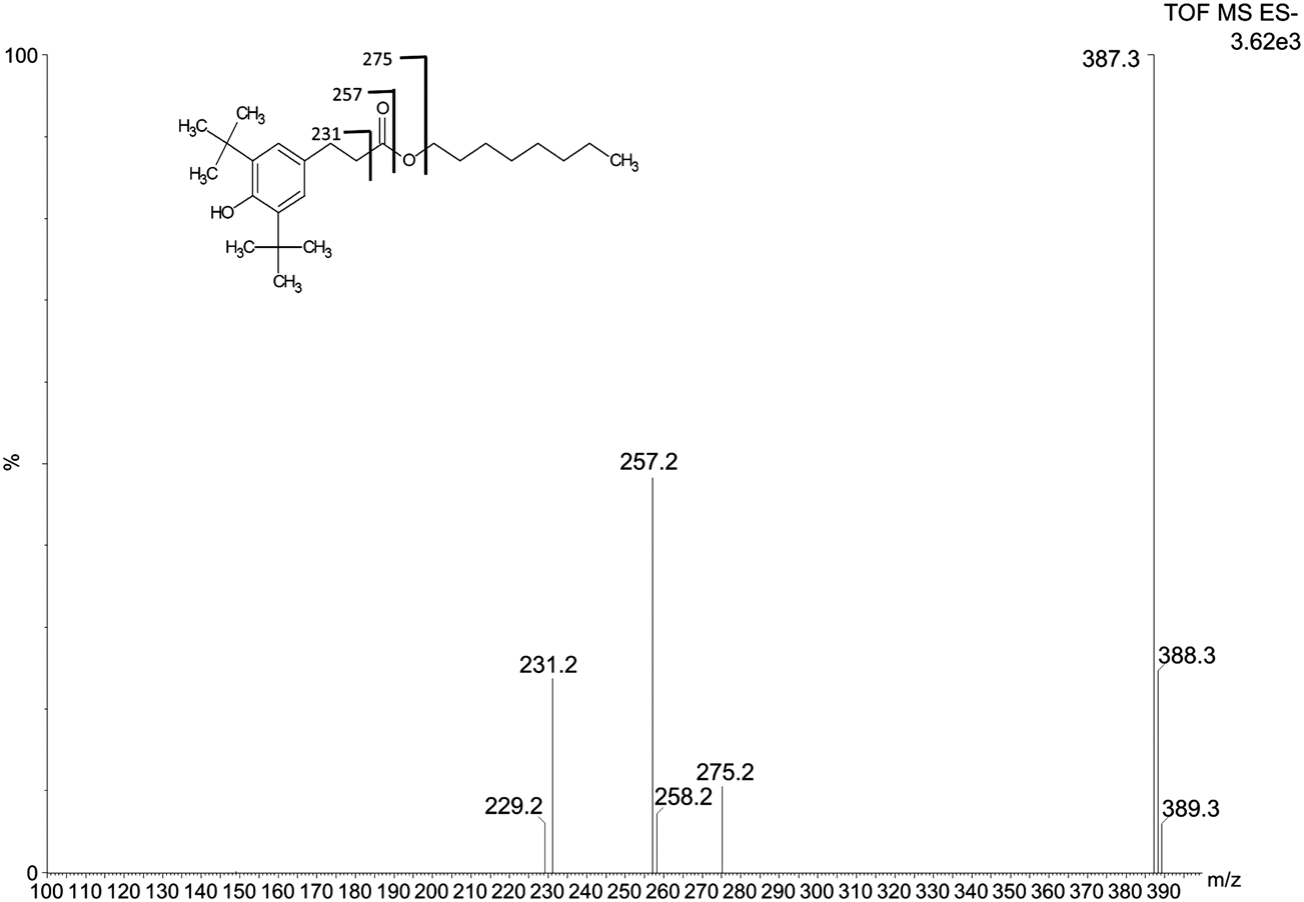
DESI-MS/MS of the [M–H]^–^ ion of **I** in an oil matrix from a filter paper surface.

DESI-MS/MS was used to confirm the identity of **I** at *m*/*z* 389.3 in the lubricant oil matrix. Analyses were conducted for a standard sample of **I** in MeOH deposited on a filter paper surface and for a sample of **I** spiked into the base oil matrix and spotted onto the surface. The [M–H]^–^ ion of **I** generated from the oil matrix was isolated by the quadrupole and fragmentation induced in the trap ion guide section of the T-wave region using collision-induced dissociation (CID). The product ion spectrum of the *m*/*z* 389.3 ion of **I** (Fig. 4) observed when **I** was spiked into the base oil matrix closely matched that obtained from the CID of the standard sample of **I**, confirming the absence of interfering ions from the oil matrix. The proposed fragmentation pattern for the deprotonated molecule of **I** is shown in Fig. 4.

The quantitative determination of **I** spiked into the oil matrix was carried out by DESI-MS using the relative mass spectral response of **I**, at concentrations in the range 1–80 µg/spot, to the internal standard **II** (27 µg/spot). The calibration plot was produced by accumulating 1-min of DESI-MS data for each sample and extracting the mass spectrum. The intensities of the [M–H]^–^ ions of **I** and **II** at each concentration of **I** in the extracted mass spectra were used to calculate a ratio of **I**/**II**, which corrected for variation in the DESI ion profile (Fig. 3, insert). This generated a calibration plot (see Supplementary Fig. S1, Supporting Information) with an R^2^ value >0.993, indicating a good linear response for the DESI-MS analysis. The use of an internal standard in the analysis of **I** overcomes many inherent problems relating to the use of surface analysis techniques, such as DESI, in quantitative measurements. Inhomogeneous sample deposition on the surface and variations in the positioning and rate of movement of the sample under the electrospray during the analysis can affect the absolute response of an analyte. This is especially problematic when spotting and traversing of the sample surface are carried out manually as was the case in this experiment. Investigations into the suitability of different internal standards for quantitative surface analysis using DESI have been reported,^29^ and key properties include similarity in the solubility of the internal standard and target analyte in the spotting solutions and the electrospray solvent composition. In addition, similar proton affinities for the analyte and internal standard will reduce any potential ion suppression effects. In this study **II**, an analogue of **I**, was synthesized and used as the internal standard to minimize structural and chemical differences between the analyte and internal standard. The internal standard retains the functionality around the aromatic ring present in the target analyte, with the substitution of CH_2_ for an oxygen atom in the hydrocarbon chain (Fig. 2). This structural modification is considered to have minimal impact on the physical and chemical properties of the molecule, such as the proton affinity, making **II** a suitable internal standard. This is supported by the good precision for the DESI-MS analysis achieved by addition of the internal standard (**II**) to the spotting solution. Replicate analyses (n = 6) were conducted at each concentration to determine the precision of the technique, generating relative standard deviations (RSDs) in the range of 3–14%, which is consistent with other reported data for quantitative DESI-MS.^12^,^13^,^29^ The RSD for the additive **I** in the absence of the internal standard was in the range of 15–44%.

The limit of detection (LOD) was calculated as the blank response of **I** plus three standard deviations of the blank using the absolute mass spectral response of **I**, obtained from a section of the spot on the surface which was then related to the total spot size. The LOD was determined to be 11 ng/mm^2^ additive on spot, which relates to less than 0.7 µg ablated from the surface during the acquisition. This corresponds to <0.03% w/v, which is below the reported concentration of additives in commercial lubricant products that are typically in the range of 0.1–5% w/v. The DESI-MS method is therefore sensitive enough to detect and quantify commercial additives in a native oil matrix.

## CONCLUSIONS

The application of DESI-MS to the quantitative surface analysis of a lubricant antioxidant additive in a complex oil lubricant matrix has been demonstrated with good linearity and repeatability (R^2^ >0.99, RSD = 3–14%). Modification of the electrospray source on the Waters Synapt HDMS instrument enabled the development of a robust DESI-MS system capable of non-proximal DESI-MS analysis of surfaces. The use of an internal standard minimized variations between DESI-MS runs caused by inhomogeneous sample distribution on the surface and other factors affecting the use of DESI-MS in quantitative measurements. The LOD for the additive in the oil lubricant was below the typical levels of additive concentration found in commercial lubricant products. The reported DESI-MS procedure has the potential for the quantitative determination of sub-microgram quantities of compounds deposited on a surface in the presence of a complex oil lubricant matrix with the appropriate choice of internal standard.
