# Supplementary figures and images for: The quantitative surface analysis of an antioxidant additive in a lubricant oil matrix by desorption electrospray ionization mass spectrometry
Source: Rapid Commun Mass Spectrom. 2013 Oct 1;27(21):2420–4. doi: 10.1002/rcm.6690 (PMC3824236; doi:10.1002/rcm.6690)

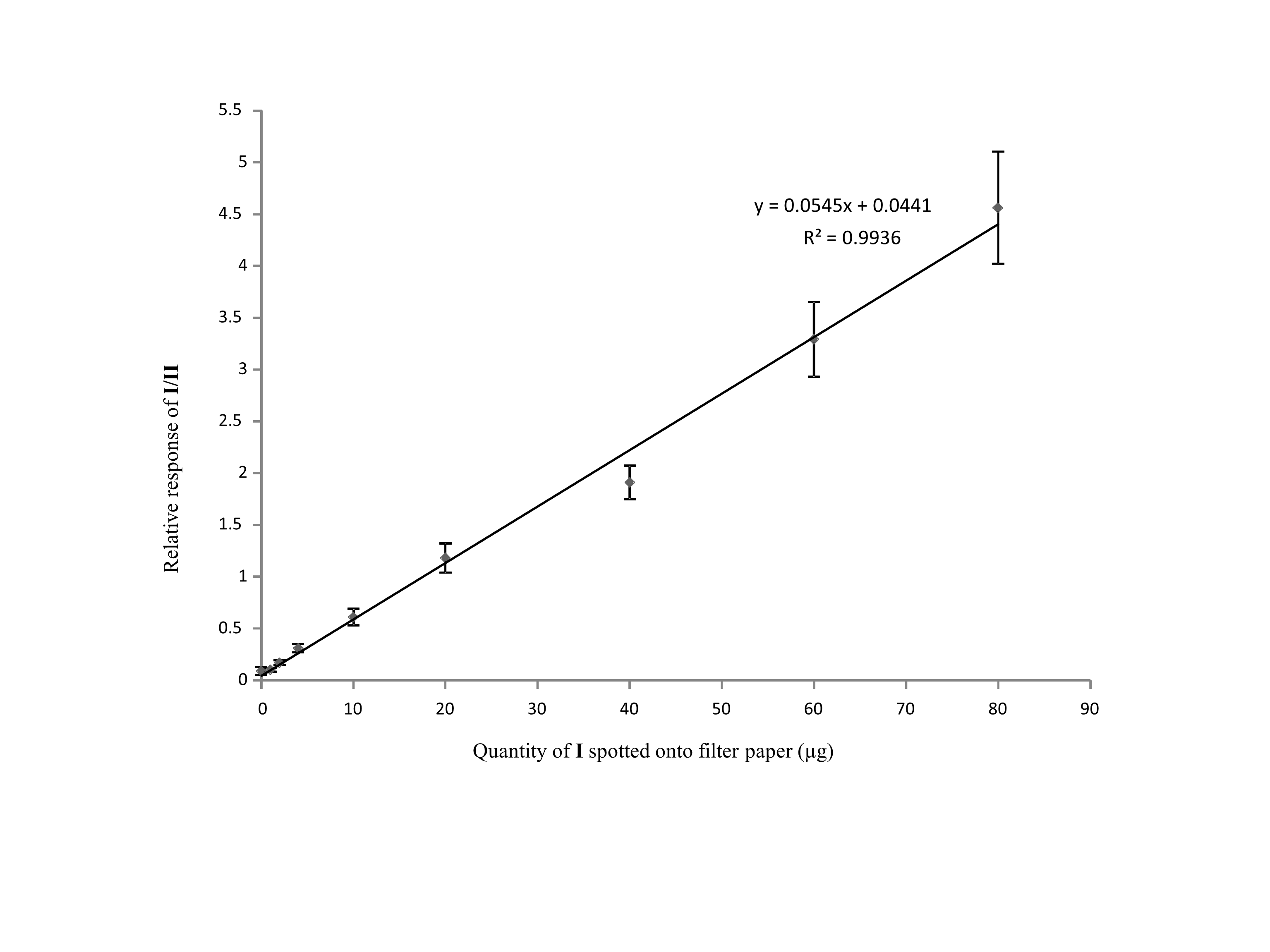

Supplement: Supplementary file 1 [file rcm0027-2420-SD1.tif]
